# Positions, Guidelines and Standardizations. Vehicles of Assistance to Medical Practice

**DOI:** 10.5935/abc.20170133

**Published:** 2017-10

**Authors:** Antônio Carlos Sobral Sousa, Claudio Pereira da Cunha, Lucélia Batista Neves Cunha Magalhães, Sergio Emanuel Kaiser, José Francisco Kerr Saraiva

**Affiliations:** 1 Departamento de Medicina e Núcleo de Pós graduação em medicina da Universidade Federal de Sergipe, Aracaju, SE; - Brazil; 2 Centro de Ensino e Pesquisa da Fundação São Lucas, Aracaju, SE; - Brazil; 3 Universidade Federal do Paraná, Curitiba, PR; - Brazil; 4 Universidade Federal da Bahia, Slavador, BA; - Brazil; 5 Universidade do Estado do Rio de Janeiro, Rio de Janeiro, RJ; - Brazil; 6 Pontifícia Universidade Católica de Campinas (PUC-Camp), São Paulo, SP - Brazil

**Keywords:** Evidence-Based Practice, Delivery of Health Care, Practice Guidelines as Topic

In a field as complex and rapidly changing as cardiology, Clinical Practice Guidelines are important tools for applying evidence-based medicine to patient care. It should be noted, however, that adherence to them varies widely, and that some doctors have concerns that these instruments characterize a rigid or simplified practice of medicine. Therefore, the appropriate implementation of health care guidelines is of great interest to national organizations, professional societies, health care providers, policy makers, legal field of medicine, patients and the public. Given the importance of the theme, several tools have been developed to assess the credibility of existing guidelines,^1^ and guidances have been elaborated step-by-step for the implementation of a practical and reliable document.^2^

Since 1992, the Brazilian Society of Cardiology (SBC) has systematically published guidelines on the most relevant themes of the specialty.^3^ However, there was a lack of discernment regarding three important concepts^4^ in the intention of the departments that integrate the SBC to carry out guidelines: a) "Guideline" - a term that should be reserved for the document that formally summarizes the evidences in the areas of diagnosis and therapeutics of pathologies; b) "Communication" (or "Standardization") shall be used for manuscripts reporting the laboratory methodology and definitions of clinical outcome and, c) "Clinical Guidance" (or "Positioning") - which should be used for official printouts that provide expert advice on challenges in patient management.

It is imperative that the documents issued by SBC should be presented with adequate titration and background to avoid confusing the reader in the differentiation of terms and, consequently, disinterest in reading them. Therefore, the main objective of this publication is to establish in a simplified and objective way, the meaning of these terminologies, aiming to standardize the issuance of Guidelines, Communications and Guidances by SBC.

## Positioning document

These documents aim to address a particular topic (diagnostic, therapeutic or laboratory) of recognized clinical interest, for which there are no (or unlikely to exist) evidence of substantial quality or, especially, those from randomized clinical trials. These documents are complementary to the guidelines and are prepared by a team of professionals with established experience in the subject.

As an example, we could cite the use of direct anticoagulants in pregnant patients.^5^ In general, the guidelines contained in these documents are anchored in the best evidence available; however, often incorporate the personal opinion of specialists.

## Clinical Guideline

Clinical guideline consists of systematically developed assertions to assist health professionals and patients in making decisions about the most appropriate form of health care under specific conditions.^6^ Unlike a guidance document, a guideline addresses a topic where there is evidence of moderate to high quality, usually from randomized trials with a satisfactory number of members, to convey the most appropriate clinical practices.

In its elaboration, a process is used to summarize the evidences (that is, systematic review) and to provide a standardized method to express the degrees of recommendations with their respective levels of evidences. To produce a guideline, it is recommended that a rigorous checklist of 146 items be followed.^2^

Therefore, these documents rarely address medical practice where evidence is scarce. They are designed to support decision-making processes in patient care; its content is based on a systematic review of clinical evidence.

## Normative document

These devices differ from those listed above since they address topics primarily focused on the standardization of clinical, laboratory and research methodologies. As an example, we could cite the Subcommittee on Anticoagulation Control of the International Society of Thrombosis and Hemostasis to measure the anticoagulant activity of factor Xa inhibitors.^7^ Therefore, it is a useful tool available to SBC departments.

The movement toward evidence-based health care has been rising rapidly in recent years, motivated by clinicians, policymakers and managers concerned about the quality, consistency, and cost of health care.

Thus, the above-mentioned documents, based on standardized best practices, provided they are written in a practical and objective manner, may be able to promote improvements in the quality and consistency of health care.
